# Morphotype-Specific Antifungal Defense in *Cacopsylla chinensis* Arises from Metabolic and Immune Network Restructuring

**DOI:** 10.3390/insects16050541

**Published:** 2025-05-20

**Authors:** Jiayue Ji, Xin Gao, Zengli Hu, Ruiyan Ma, Longlong Zhao

**Affiliations:** 1 Institute of Pomology, Shanxi Agricultural University, Taiyuan 030031, China; jijiayue1991@163.com (J.J.); gsshuzengli@163.com (Z.H.); 2 College of Plant Protection, Shanxi Agricultural University, Jinzhong 030801, China; gx15534355877@163.com (X.G.); maruiyan2019@163.com (R.M.)

**Keywords:** *Cacopsylla chinensis*, *Beauveria bassiana*, seasonal polyphenism, transcriptomic response

## Abstract

Pear psylla (*Cacopsylla chinensis*), a major pear tree pest widely distributed in China, is increasingly affecting the productivity of orchards. This species exhibits seasonal polyphenism with two distinct forms, namely, a summer form and a winter form. Through topically applying *Beauveria bassiana* conidial suspensions to the abdominal cuticle of *C. chinensis*, we demonstrated that the entomopathogenic fungus *B. bassiana* exhibits significant yet phenotypically divergent virulence against these two forms. Using PacBio SMRT sequencing and Illumina RNA-seq, we analyzed transcriptomic changes post-infection, revealing form-specific immune responses, with 18,232 and 5027 differentially expressed genes identified in summer- and winter-form pear psylla, respectively, and a total of 3715 DEGs shared between the two seasonal phenotypes. In summer-form individuals, *B. bassiana* infection disrupted oxidative phosphorylation and downregulated immune recognition genes, cellular immune-related genes, and signaling genes, along with the upregulation of the immune inhibitor serpin, indicating immunosuppression. Conversely, in winter-form individuals, immune-related genes and glycolytic rate-limiting enzymes were upregulated after infection, suggesting that the winter-form immune system normally responds to *B. bassiana* infection and supports efficient defense through metabolic reprogramming to fuel energy-demanding defenses. These findings advance our understanding of *C. chinensis*/*B. bassiana* interactions, providing a basis for elucidating immune regulation in seasonally polymorphic insects. The results also inform strategies to optimize *B. bassiana*-based biocontrol, contributing to sustainable pear psylla management.

## Simple Summary

Pear psylla, a major pest in Chinese pear orchards, has two seasonal forms: summer and winter. We investigated why the entomopathogenic fungus *Beauveria bassiana* affects both forms differently. Transcription analysis revealed that when summer-form pear psylla were exposed to *B. bassiana*, their immune systems were suppressed. In contrast, winter-form pear psylla enhanced immune responses and made energy adjustments to resist *B. bassiana* effectively. This explains the winter form’s stronger resistance. These results advance our understanding of the immunological mechanisms underlying seasonal polyphenism and suggest that fungal biopesticides could be enhanced by developing immune-suppressing adjuvants (e.g., RNA interference-targeting immunity genes or biochemical inhibitors of key immune enzymes). Such precision strategies could synergize with fungal pathogens to achieve higher pest mortality rates, enabling substantially reduced chemical pesticide applications while maintaining effective control. This approach provides a targeted biological control framework that offers sustainable agricultural solutions by minimizing environmental contamination and delaying the evolution of pesticide resistance.

## 1. Introduction

Pears are among the most economically significant fruit crops worldwide, with China being the leading producer and exporter (FAO 2022). *Cacopsylla chinensis* (Yang and Li) (Hemiptera: Psyllidae) is the primary pest affecting pear orchards in China, and its prevalence is increasing annually [1,2]*. C. chinensis* feeds on phloem sap, leading to defoliation, reduced tree vigor, and fruit quality degradation [3]. Additionally, honeydew secretion interferes with predation and facilitates sooty mold growth, further obstructing photosynthesis and affecting fruit marketability [4]. *C. chinensis* exhibits seasonal polyphenism, with its summer-form and winter-form phenotypes differing in size, coloration, cold tolerance, hunger tolerance, and dispersal ability. The unique biological characteristics of *C. chinensis* complicate relevant pest control efforts, and extensive pesticide use has led to the evolution of high resistance in this species [5,6]. As *C. chinensis* has not yet spread to neighboring countries such as South Korea and Japan, it is critical to develop efficient and environmentally friendly control strategies to ensure agricultural sustainability and biosecurity [2,7].

Biological control is environmentally friendly, sustainable, and has low energy consumption, which is conducive to the green production of fruit. However, under natural conditions, the effectiveness of biological control, such as the use of *Psylladintus insidiosus*is, an obligate parasitic natural enemy, is often not ideal [8]. *Beauveria bassiana* is a broad-spectrum invertebrate fungal pathogen that has been utilized in the development of diverse commercial products and successfully used in the control of various pests, such as *Bemisia tabaci*, *Gonipterus scutellatus*, *Hypothenemus hampei*, and *Lycorma delicatula* [9,10,11,12]. Previously, we found that *Beauveria bassiana* has the ability to infect *C. chinensis* in the field and isolated a *B. bassiana* strain, GSSBb1901. However, the control ability of *B. bassiana* against different phenotypes of *C. chinensis* remains unclear. Considering the higher susceptibility of the summer form compared with the winter form to chemical pesticides during application, we hypothesized that the two phenotypes of *C. chinensis* may also exhibit differential susceptibility to *B. bassiana*. Studies have shown that insects can rely on a variety of physiological responses, including their innate immune system, for self-protection, and pathogenic microorganisms have also evolved a variety of modes to improve virulence [13]. Therefore, the study of the response of two phenotypes of *C. chinensis* to *B. bassiana* is helpful to understand the host/pathogen interaction, phenotype-specifically optimize biological control strategies, and, ultimately, enhance *B. bassiana*’s field efficacy for the management of *C. chinensis*. The innate immune system in insects is relatively conservative and can be divided into three main steps: First, pattern recognition proteins (PRPs) detect pathogen-associated molecular patterns (PAMPs) to start the immune process. Second, serine proteases (SPs) take over. Once the PRPs have found the PAMPs, they adjust and pass on the immune signal. Third, hemocytes and fat body cells produce immune-related molecules (such as antimicrobial peptides (AMPs)) to fight off the invading microorganisms [14,15,16]. However, there are still complex differences in the immune systems between different species, such as the type of hemocytes, the function of immune genes, and the regulating mode [15,17,18,19]. At present, knowledge about the immune system of *C. chinensis* is very limited due to a lack of genome and transcriptome information.

With the advancement of molecular biology techniques, short-read transcriptome sequencing has become a common tool for the description of gene expression levels [20,21]. However, for species without genomic information, this method cannot provide complete and accurate information about the transcriptome [22]. Thus, third-generation sequencing (TGS) technology was created to obtain full-length transcripts [23,24]. In this study, we combined single-molecule real-time (SMRT) sequencing and next-generation sequencing (NGS) techniques to explore the immune response in *C. chinensis* stimulated by *B. bassiana* and any phenotype-specific differences. The results provide a good basis for further research on the immune system of *C. chinensis*, deepen our understanding of the physiological changes in multi-phenotypic insects, and provide valuable resources regarding the use of fungi to control pests.

## 2. Materials and Methods

### 2.1. Insect and Fungi

*C. Chinensis* (summer form) was originally collected from Shanxi Agricultural University, the Pomology Institute (37°20′35″ N, 112°29′32″ E; Taigu County, Shanxi Province, China), and fed on branches of *Pyrus bretschneideri* cultivar ‘Yu lu xiang’ pears at 20 °C with 60–80% relative humidity and a 12L–12D photoperiod. To obtain the winter-form *C. chinensis*, the *C. chinensis* samples were raised from the egg stage under conditions of 15 °C and a 16L–8D photoperiod. After they reached adulthood, the rearing conditions were consistent with those of the summer form. *B. bassiana* GSSBb1901 was isolated in our laboratory from *B. bassiana-*infected *C. chinensis* in the field and was cultured on potato dextrose agar plates at 25 °C in 60–80% relative humidity. Conidia were harvested from 4-week-old cultures and diluted to a final concentration of 2.5 × 10^7^ spores/mL in sterile phosphate-buffered saline (PBS).

### 2.2. Experimental Design

To assess fungal pathogenicity, two seasonal phenotypes of *C. chinensis* were subjected to topical inoculation with 1 μL of *B. bassiana* conidial suspension (2.5 × 10^6^ spores/mL, 2.5 × 10^7^ spores/mL, or 2.5 × 10^8^ spores/mL) or a PBS control. The solution was applied to the abdomen using a pipette. Mortality was monitored at 24 h intervals, with survival Kaplan–Meier curves generated through daily observations. Each treatment group consisted of 80 individuals.

To calculate the changes in the hemocyte concentration, the hemolymphs of the treated *C. chinensis* (2.5 × 10⁷ spores/mL or the PBS control) at 24 h post-infection (hpi) were collected by cutting their heads. The collected hemolymphs (0.1 μL) were immediately diluted in ice-cold PBS (1:10 *v*/*v*) and loaded into a hemocytometer chamber for cell counting. Each treatment included six samples, and each sample comprised two *C. chinensis*, with technical duplicates performed per sample.

For transcriptome analysis, infected *C. chinensis* (2.5 × 10^7^ spores/mL or the PBS control) were sampled at 48 hpi and frozen in liquid nitrogen. Three biological replicates (0.15 g per replicate, with about 250 individuals) per treatment were subjected to Illumina sequencing, SMRT-Seq, and quantitative real-time PCR (qRT-PCR) validation.

### 2.3. RNA Extraction and Sequencing

Total RNA was extracted using TRIzol reagent (Invitrogen, Carlsbad, CA, USA), with the purity and concentration assessed using a Nanodrop 2000 spectrophotometer (Thermo Fisher Scientific, Waltham, MA, USA), Agilent 2100, and LabChip GX (Perkin-Elmer, Waltham, MA, USA).

The total RNA from 12 samples was mixed equally for the PacBio SMRT-Seq library construction. The SMRTbell Template Prep Kit, PacBio Binding Kit, and AMpure PB Beads (Pacbio, Menlo Park, CA, USA) were used for the repair, binding, and purification of the mixed samples. The final products were sequenced on a Sequel II platform (Pacbio, Menlo Park, CA, USA). The Illumina sequencing library was constructed using the Hieff NGS Ultima Dual-mode mRNA Library Prep Kit and Hieff NGS DNA Selection Beads (Yeasen, Shanghai, China) and was sequenced on an Illumina NovaSeq 6000 platform (Illumina, San Diego, CA, USA). The above sequencing was performed at Biomarker Technologies (Beijing, China).

### 2.4. Data Processing and Analysis

The raw subreads of the SMRT sequencing were analyzed to generate high-quality full-length non-redundant transcript (FLNRT) consensus sequences using Iso-Seq3 v3.4.0 and CD-HIT v4.6.1 [25]. For the Illumina sequencing, adapter contamination, poly-N contamination (proportion of N > 10%), and low-quality reads (over 50% of bases showing Q ≤ 10 in the quality value) in the raw reads were filtered to obtain clean reads. All the downstream analyses were based on high-quality, clean data.

### 2.5. Gene Functional Annotation

TransDecoder version 5.0.0 was used to predict the coding sequences (CDSs) and amino acid sequences of the FLNRTs.

The functions of the FLNRTs were annotated based on six databases: the NR (NCBI non-redundant protein sequences) database, Pfam (Protein family) database, GO (Gene Ontology) database, KEGG (Kyoto Encyclopedia of Genes and Genomes) database, eggNOG (Evolutionary Gene Genealogy: Non-supervised Orthologous Groups) database, and Swiss-Prot (a manually annotated and reviewed protein sequence database) database. Software such as Diamond v2.0.15 [26], InterProScan v5.34-73.0 [27], and Hmmscan v3.3.2 were used in this process.

### 2.6. Differentially Expressed Genes (DEGs) Analysis and Enrichment Analysis

To understand the gene expression levels of *C. chinensis* under fungal stress, the FLNRTs yielded from the SMRT sequencing were used as the reference transcriptome, and the Illumina clean reads were mapped to this reference transcriptome for quantitative analysis using STAR v2.5.0b [28] and Kallisto v0.46.1 [29]. To eliminate the effect of the sequencing depth and transcript length, the gene transcription levels were estimated by the fragments per kilobase of transcript per million fragments mapped (FPKM). Differential expression analysis between the fungal infection group and control group was performed using edgeR v5.0.0 [30]. To control the influence of false positives on the results, the Q-values were adjusted to obtain false discovery rate (FDR) values with Benjamini–Hochberg’s procedure. In the DEG detection process, |log2 Fold Change| ≥ 1 and FDR ≤ 0.01 were used as screening criteria for DEGs. To further analyze the functions of the DEGs, GO and KEGG enrichment analyses were performed using the clusterProfiler v4.4.4 [31] package.

### 2.7. Quantitative Real-Time PCR Analysis

Eight genes were randomly selected to validate the accuracy of the transcriptome data. The qPCR was performed with a CFX96 Touch Real-Time PCR Detection System (Bio-Rad, Hercules, CA, USA) using 2 × qPCR SYBR Master Mix (Tolo Biotech, Shanghai, China), according to the manufacturer’s instructions. The specific primers are listed in Table S1, and *rPL45* was used as an internal standard to normalize the expression level. The relative expression levels were calculated using the 2^−ΔΔCt^ method.

### 2.8. Statistical Analysis

Statistical analyses were performed using GraphPad Prism version 6.0. Survival curves were calculated by employing the Kaplan–Meier method, and the differences between them were evaluated using the log-rank (Mantel–Cox) test (*p* < 0.0001). Significance testing of the changes in the hemocyte concentration was performed using an unpaired two-tailed *t*-test (*p* < 0.05).

## 3. Results

### 3.1. Summer-Form C. chinensis Was More Sensitive to B. bassiana Than Winter-Form C. chinensis

**Figure 1:**
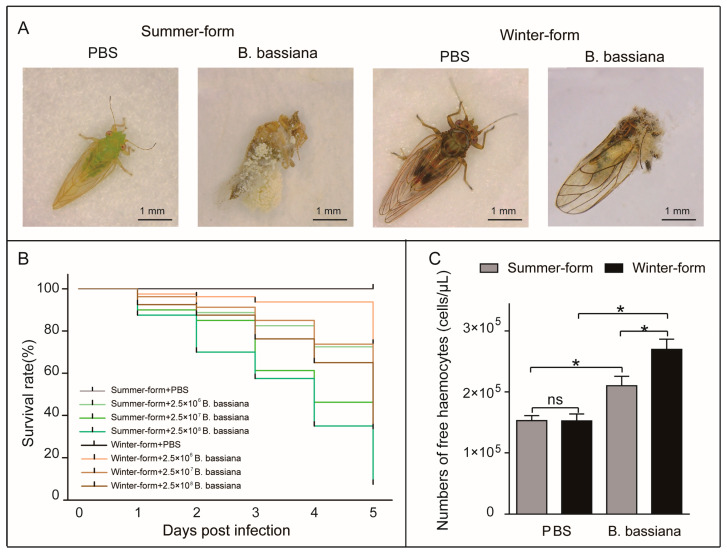
Effects of *B. bassiana* on two phenotypes of *C. chinensis*. (**A**) Images of *C. chinensis* individuals infected with *B. bassiana* conidia or PBS. (**B**) Survival curves of *C. chinensis* infected with *B. bassiana* conidia (2.5 × 10^6^, 2.5 × 10^7^, and 2.5 × 10^8^ conidia per individual) or PBS (*n* = 80). The differences were evaluated by using the log-rank (Mantel–Cox) test (*p* < 0.0001). (**C**) Cell concentration of *C. chinensis* infected with *B. bassiana* (2.5 × 10^7^ conidia per individual), and the cellular density of the hemocytes was determined using a hemocytometer chamber. The bars represent the means ± SEM (*n* = 6). “ns” indicate no significant difference. The asterisks indicate significant differences (unpaired *t*-test; *p* < 0.05).

The fungus *B. bassiana* infected *C. chinensis*. After infection, the mobility of *C. chinensis* declined progressively until death, and *B. bassiana* hyphae covered the body three days after the insect died (Figure 1A). With an increase in *B. bassiana* exposure, the *C. chinensis* survival time decreased. Among the samples, the winter form, which infects equal amounts of *B. bassiana*, survived significantly longer than the summer form (*p* < 0.0001) (Figure 1B). In addition, we identified six types of circulating hemocytes in the hemolymph of the *C. chinensis*, including prohemocytes, plasmatocytes, oenocytoids, granulocytes, spherulocytes, and megakaryocytes (Supplementary Figure S1). After infection with *B. bassiana*, the number of free hemocytes in the two seasonal phenotypes of *C. chinensis* increased. Moreover, the number of free hemocytes of *B. bassiana-*infected winter-form *C. chinensis* was more than that of *B. bassiana-*infected summer-form *C. chinensis* (Figure 1C).

### 3.2. Overview of PacBio and Illumina Sequencing

In this study, 12 samples of two forms of *C. chinensis* under fungal infection conditions were mixed to construct the PacBio SMRT-Seq library, and a comprehensive transcription profile was obtained. A total of 116,535 circular consensus sequencing (CCS) reads with an average length of 1743 bp and 93,175 full-length non-chimeric reads (FLNCs) were obtained after quality control. Following classification, a total of 35,503 high-quality isoforms and five low-quality isoforms (with a mean length of 1724 bp) were identified. A total of 30,097 full-length non-redundant transcripts (FLNRTs) were assembled for a follow-up study (Table 1). Using Illumina sequencing, each sample produced 21–27 million clean reads, with a Q30 quality score exceeding 94%, indicating a high sequencing accuracy.

### 3.3. Functional Annotation and Classification

**Figure 2:**
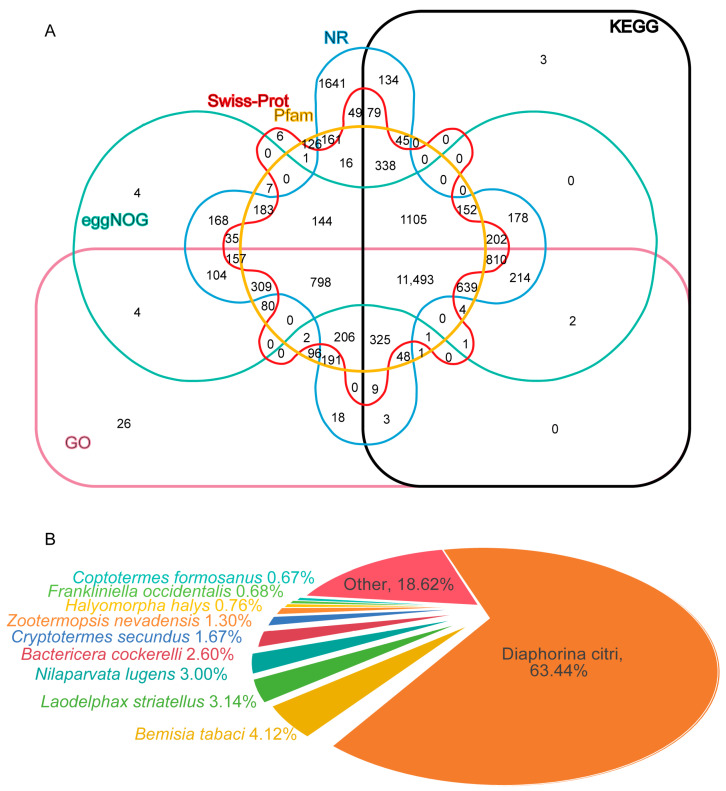
Unigene annotation of PacBio sequencing. (**A**) Venn diagrams of all unigenes according to six databases. (**B**) Homologous species distribution annotated based on the NR database.

To predict the comprehensive functions of *C. chinensis* transcripts, we annotated them using the NR, GO, KEGG, Pfam, eggNOG, and Swiss-Prot databases. The results show that, in total, 20,318 transcripts were annotated in at least one database, and 11,493 transcripts were annotated in all six databases. In detail, the highest number of unigene hits was found in the NR database, followed by the GO, eggNOG, Pfam, KEGG, and Swiss-Prot databases, with 19,695, 17,530, 16,622, 16,471, 15,579, and 12,844 annotated transcripts (Figure 2A). Based on NR annotations, 12,494 (63%) of the *C. chinensis* transcripts were aligned to *Diaphorina citri*, followed by *Bemisia tabaci* (812 (4%)), *Laodelphax striatellus* (619 (3%)), *Nilaparvata lugens* (590 (3%)), *Bactericera cockerelli* (513 (3%)), *Cryptotermes secundus* (329 (2%)), *Zootermopsis nevadensis* (256 (1%)), *Halyomorpha halysand* (149 (1%)), *Frankliniella occidentalis* (134 (1%)), *Coptotermes formosanus* (132 (1%)), and others (3667 (19%)) (Figure 2B). A total of 126 transcripts with high similarity to immune-related genes were identified, comprising 19 transcripts for microbial recognition, 47 transcripts for signal transduction, and 60 transcripts for effector production (Table S2).

**Figure 3:**
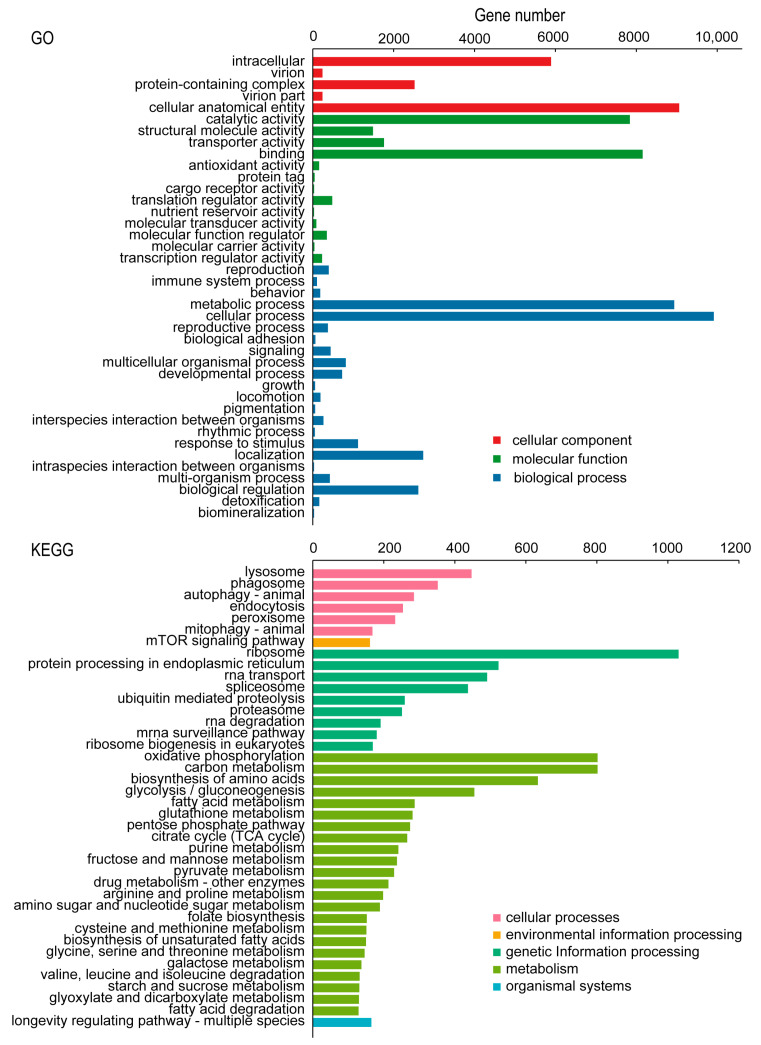
GO and KEGG classification of *C. chinensis* transcripts.

In the GO classification, the transcriptome of *C. chinensis* was clustered into 56 subcategories of the 3 major categories (cellular component, molecular function, and biological process). In the cellular component category, the second-level classification of cellular process (1411 transcripts) and metabolic process (1268 transcripts) had the most transcripts. Regarding the molecular function category, the most abundant subcategories were cellular anatomical entity (9059 transcripts) and catalytic activity (7389 transcripts). In the biological process category, cellular process (9915 transcripts) had the largest number of transcripts, followed by metabolic process with 8938 transcripts and localization with 2731 transcripts. In the KEGG classification, the transcripts were annotated into 277 KEGG pathways, which were grouped into 6 major categories (cellular processes, environmental information processing, genetic information processing, metabolism, organismal systems, and human diseases) and 44 subcategories. With respect to the cellular processes category, lysosome (447 transcripts) was the main subcategory. For environmental information processing, the mTOR signaling pathway (161 transcripts) had the most transcripts. In the genetic information processing category, ribosome (1030 transcripts) was the largest subcategory. For metabolism, the two second-level classifications of oxidative phosphorylation and carbon metabolism contained the most transcripts, each containing 802 transcripts. In the organismal systems category, the largest number of transcripts was assigned to the longevity-regulating pathway—multiple species (165 transcripts). In the human diseases category, herpes simplex virus 1 infection (27 transcripts) was the largest group subcategory (Figure 3).

### 3.4. Identification of DEGs

**Figure 4:**
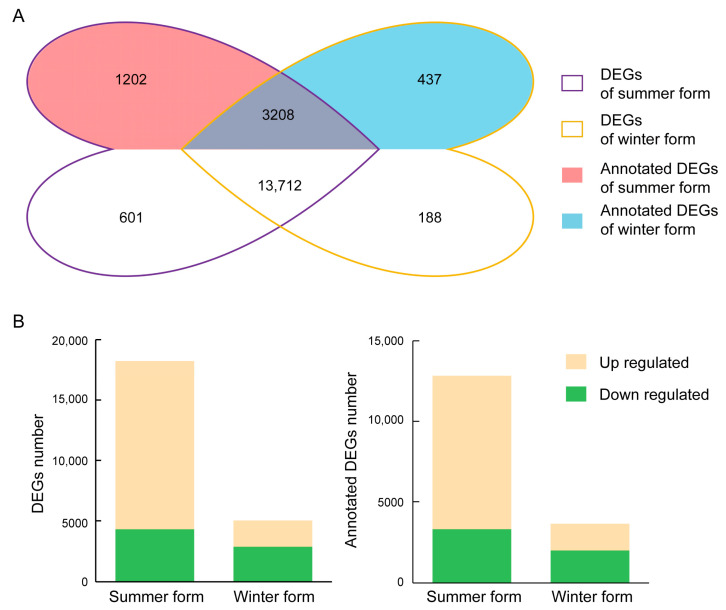
Overview of the DEGs of C. chinensis upon B. bassiana infection. (**A**) Venn diagrams of DEGs in two seasonal phenotypes. (**B**) Numbers of DEGs induced in two seasonal phenotypes.

To identify DEGs responding to *B. bassiana* infection, we analyzed two seasonal phenotypes of *C. chinensis*. After fungal infection, 18,232 and 5027 DEGs were identified in the summer form and winter form, respectively, and a total of 3715 DEGs were shared between the two seasonal phenotypes. In detail, in the summer form, 76% of DEGs were upregulated, and 24% of DEGs were downregulated. In the winter form, 43% of DEGs were upregulated, and 57% of DEGs were downregulated. There were 12,832 and 3641 DEGs annotated in at least one database in the summer form and winter form, respectively. A total of 2789 DEGs were identified in both seasonal phenotypes. Among them, 876 DEGs were upregulated and 339 DEGs were downregulated in both seasonal phenotypes (Figure 4).

### 3.5. DEGs in Response to B. bassiana Infection

**Figure 5:**
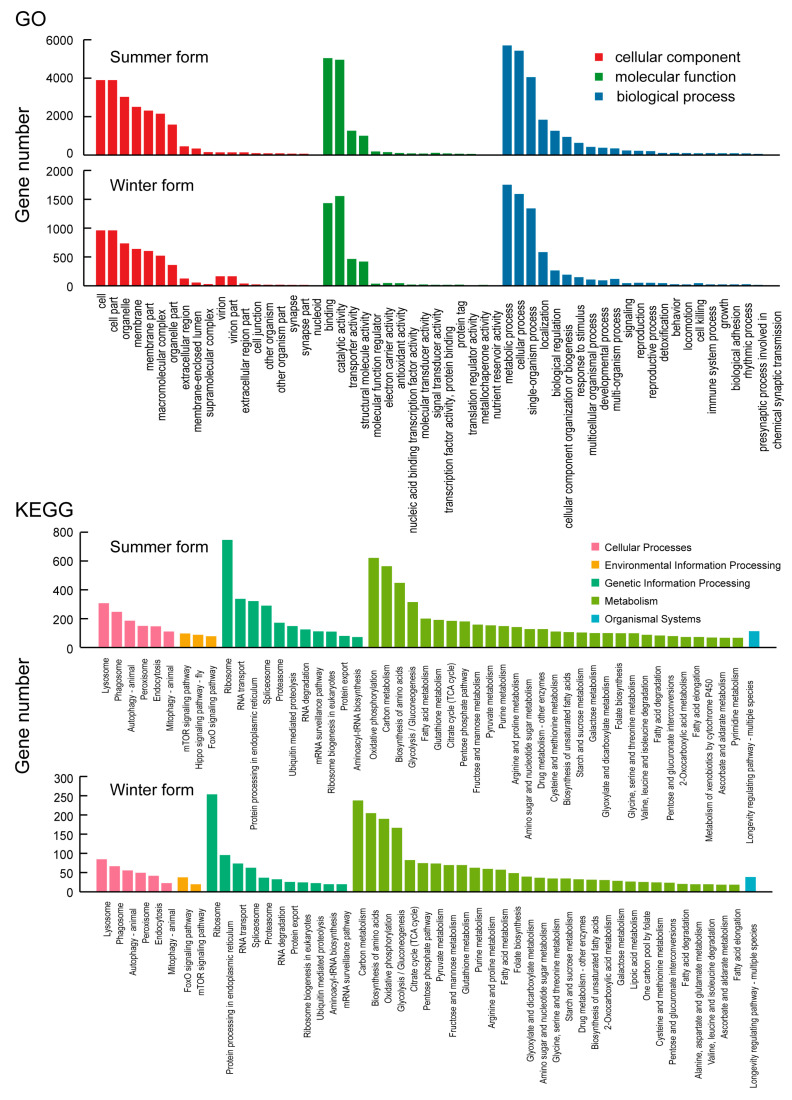
GO and KEGG classification of *C. chinensis* upon *B. bassiana* infection.

In the GO classification, the DEGs in summer-form and winter-form *C. chinensis* were classified into 53 subcategories each. For the KEGG classification, the DEGs were divided into 45 and 40 subcategories, respectively. Among them, most of the subcategories and DEGs belonged to the major categories of metabolism and genetic information processing. In detail, translation was the subcategory with the largest number of DEGs. In cellular processes, lysosome and peroxisome had the largest numbers of DEGs in the two treatment processing groups. In environmental information processing, the mTOR signaling pathway and FoxO signaling pathway were the most abundant KEGG pathways in the summer form and winter form, respectively. In genetic information processing, ribosome had the largest number of DEGs in the two treatment processing groups. For metabolism, oxidative phosphorylation, carbon metabolism, biosynthesis of amino acids, and glycolysis/gluconeogenesis were the pathways with the most DEGs in the summer and winter forms. In organismal systems, the longevity-regulating pathway—multiple species had the largest number of DEGs in the two treatment processing groups (Figure 5; Supplementary Table S3).

**Figure 6:**
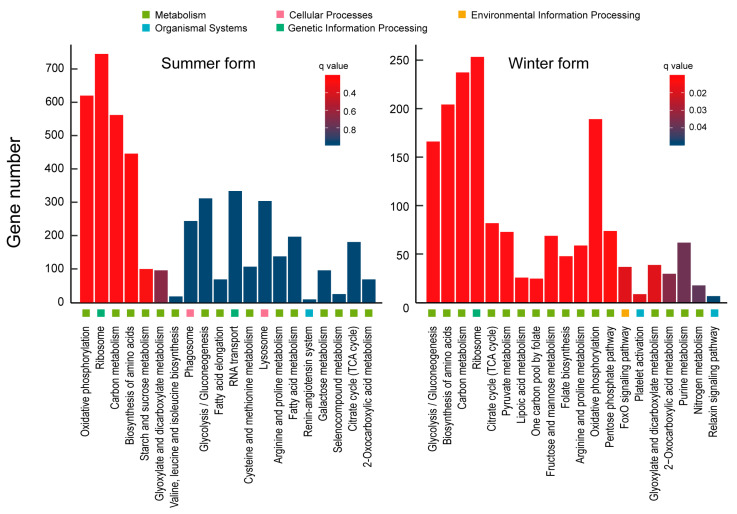
KEGG enrichment analysis of *C. chinensis* upon *B. bassiana* infection.

The results of the KEGG enrichment showed that oxidative phosphorylation and glycolysis/gluconeogenesis were the most significant categories in the summer form and winter form, respectively. Oxidative phosphorylation, ribosome, and carbon metabolism were highly enriched pathways in the summer form. Glycolysis/gluconeogenesis, biosynthesis of amino acids, carbon metabolism, ribosome, citrate cycle (TCA cycle), pyruvate metabolism, lipoic acid metabolism, one-carbon pool by folate, fructose and mannose metabolism, folate biosynthesis, arginine, and proline metabolism, oxidative phosphorylation, pentose phosphate pathway, FoxO signaling pathway, platelet activation, glyoxylate and dicarboxylate metabolism, 2-oxocarboxylic acid metabolism, purine metabolism, nitrogen metabolism, relaxin signaling pathway, and glutathione metabolism were highly enriched pathways in the winter form (Figure 6).

### 3.6. DEGs in Two Seasonal Phenotypes of C. chinensis Infected by B. bassiana

**Figure 7:**
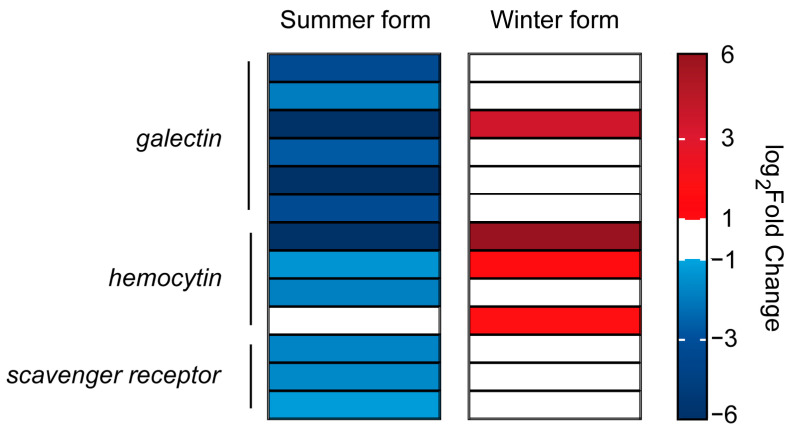
DEGs related to the pattern recognition process in *C. chinensis* infected by *B. bassiana*.

Infection by pathogenic fungi can trigger a response from the insect’s innate immune system. The recognition of pathogens is the first step in the immune response of insects. A total of 19 signal recognition genes were identified, including 2 *dscam* genes, 9 *galectin* genes, 4 *hemocytin* genes, and 4 *scavenger receptor* genes. In the summer form, 12 of the 14 DEGs were downregulated, and 4 of the 5 DEGs were upregulated in the winter form. Among them, one *galectin* and two *hemocytin* genes were downregulated in the summer form and upregulated in the winter form (Figure 7; Supplementary Table S2).

Regarding the immune signal transduction process, four clip domain-containing *SP* genes were identified. Three SP genes were identified as phenoloxidase-activating enzymes, and two SP genes were downregulated in the summer form, whereas two were upregulated in the winter form. Another clip domain-containing *SP* was identified as a *Spätzle-processing enzyme* (*SPE*) and was upregulated in the winter form and downregulated in the summer form. In addition, two *phenoloxidase* genes identified as downstream genes of *SPE* were downregulated in the summer form. Furthermore, one *Spätzle* (*spz*) and three *Toll-like receptor* genes downstream of *SPE* were identified, in which *spz* and one *Toll-like receptor* were downregulated in the summer form and upregulated in the winter form.

**Figure 8:**
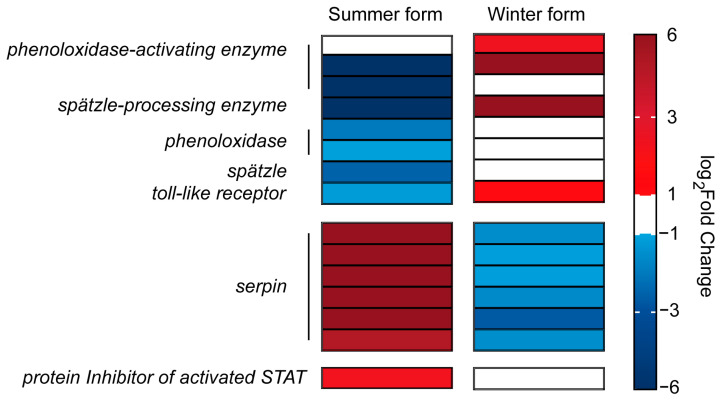
DEGs related to the signal transduction process in *C. chinensis* infected by *B. bassiana*.

The immune process needs to be critically regulated to maintain physiological balance, and inhibitors are important factors in insect homeostasis. In this study, 25 *serine protease inhibitors* (*serpins*) were identified. In the summer form, 21 of 25 DEGs were upregulated, and 7 *serpins* were downregulated in the winter form. In addition, six *serpins* were upregulated in the summer-form but downregulated in the winter-form pear psylla. Moreover, one inhibitor of the Jak/stat pathway, *protein inhibitor of activated STAT* (*PIAS*), was upregulated in the summer form (Figure 8; Supplementary Table S2).

**Figure 9:**
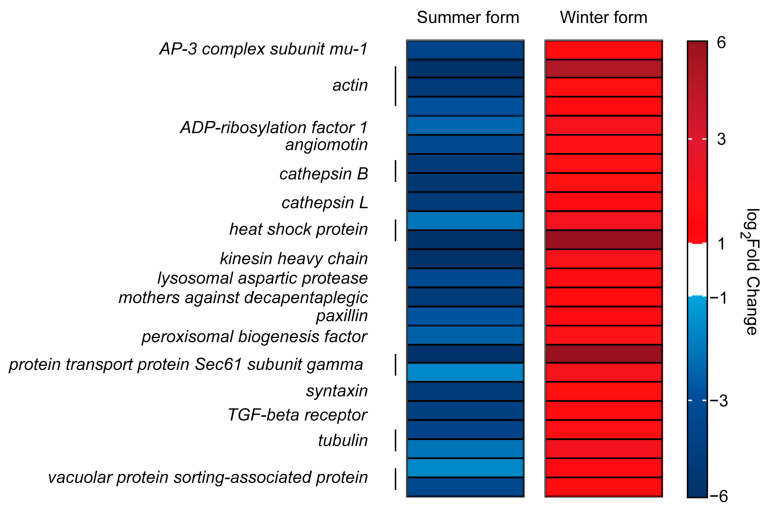
DEGs related to cellular processes in *C. chinensis* infected by *B. bassiana*.

Considering that the cell is an important immune organ, we analyzed the genes related to cellular processes. A total of 1278 genes were identified in the two groups, and 975 genes responded to *B. bassiana* infection and participated in cell growth and death, cell motility, cellular community, and transport and catabolism. Among them, twenty-four genes were downregulated in the summer form and upregulated in the winter form, including one *AP-3 complex subunit mu-1*, three *actin* genes, one *ADP-ribosylation factor 1*, one *angiomotin*, two *cathepsin B*, one *cathepsin L*, two *heat shock protein*, one *kinesin heavy chain*, one *lysosomal aspartic protease*, one *mothers against decapentaplegic*, one *paxillin*, one *peroxisomal biogenesis factor*, two *protein transport protein Sec61 subunit gamma*, one *syntaxin*, one *TGF-beta receptor*, two *tubulin,* and two *vacuolar protein sorting-associated protein* genes (Figure 9).

**Figure 10:**
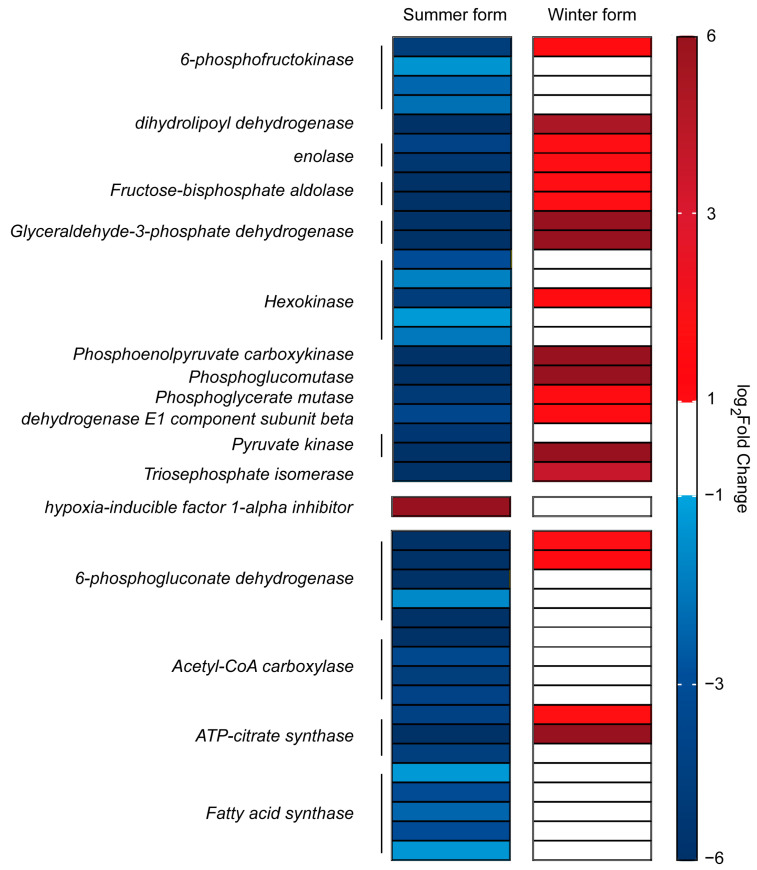
DEGs related to the metabolic pathways in *C. chinensis* infected by *B. bassiana*.

Metabolism is crucial for the maintenance of life and health of organisms and is also the most relevant category of genes in response to *B. bassiana* infection in *C. chinensis*. Among the genes associated with metabolism that were identified simultaneously in the two treatment groups (3589 transcripts), 78% (2811 transcripts) were differently expressed after *B. bassiana* infection in the summer form, and most of them were upregulated, and the most upregulated DEGs belonged to the oxidative phosphorylation pathway. However, in the winter form, most of the DEGs involved in the oxidative phosphorylation pathway were downregulated. For another important energy metabolic pathway, glycolysis/gluconeogenesis, we identified eight *hexokinase* genes and six *6-phosphofructokinas* genes. Among them, six *hexokinase* and four *6-phosphofructokinas* genes were downregulated in the summer form, while one *hexokinase* gene and one *6-phosphofructokinas* gene were upregulated in the winter form. Furthermore, the transcription levels of *dihydrolipoyl dehydrogenase*, *enolase*, *fructose-bisphosphate aldolas*e, *glyceraldehyde-3-phosphate dehydrogenase*, *phosphoenolpyruvate carboxykinase*, *phosphoglucomutase*, *phosphoglycerate mutase*, *dehydrogenase E1 component subunit beta*, *pyruvate kinase*, and *triosephosphate isomerase* also showed the regularity of downregulation in the summer form and upregulation in the winter form. One of the key regulators of glycolytic metabolism, *hypoxia-inducible factor 1-alpha inhibitor*, was upregulated in the summer form. Furthermore, for key enzymes of fatty acid biosynthesis and the pentose phosphate pathway, four of eight *acetyl-CoA carboxylase*, six of nine *fatty acid synthase*, five of seven *ATP citrate lyase*, and seven *6-phosphogluconate dehydrogenase* genes were downregulated in the summer form, and two *6-phosphogluconate dehydrogenase* genes and two *ATP-citrate synthase* genes were upregulated in the winter form (Figure 10).

### 3.7. Validation of Gene Expression Levels

Through qRT-PCR analysis, the expression trends of eight randomly selected transcripts across different treatment groups were found to be consistent with the transcriptome-sequencing results. This consistency validates the reliability of the transcriptome data (Supplementary Figure S2).

## 4. Discussion

The immune system serves as a crucial protective barrier for insects, and a deeper understanding of its molecular mechanisms can enhance pest control strategies. *C. chinensis*, a significant pear pest, exhibits phenotypic plasticity in response to environmental changes, making it a suitable model organism for studying context-dependent immune regulation. In this study, we investigated the divergent immunocompetence between summer-form and winter-form *C. chinensis* and analyzed the transcriptome with SMRT-Seq and RNA-Seq for the first time. Using RNA sequencing, a total of 30,097 FLNRTs were obtained. Upon *B. bassiana* infection, 18,232 and 5027 DEGs were identified in the summer form and winter form, respectively. In addition to genes related to the innate immune systems, these DEGs also contained genes related to metabolism, cellular processes, environmental information processing, organismal systems, and human diseases. To our knowledge, this is the first study to obtain antifungal-related genetic information about *C. chinensis* without a reference genome and at a reduced cost.

The innate immune system of insects consists of two main components: cellular immunity and humoral immunity [32,33]. Cellular immunity is mainly carried out by hemocytes and includes phagocytosis, encapsulation, and nodulation [34]. Here, we identified five types of hemocytes. Among them, it has been demonstrated that prohemocytes, plasmatocytes, oenocytoids, and granulocytes are involved in immune responses in *Drosophila*, *Armigeres subalbatus*, and *Bombyx mori* [35,36,37]. In addition, we detected many immune-related genes in *C. chinensis*, including four types of *PRPs*, and genes involved in the Toll pathway, IMD pathway, melanization process, Jak/stat pathway, and Jnk pathway. These pathways have been shown to be widespread in arthropods to transmit immune signals and induce effect products [38,39,40]. This suggests that *C. chinensis* may rely on these hemocytes and immune pathways to achieve high survival. AMPs are widely distributed in fungi, plants, and animals, functioning as active peptides against pathogens such as fungi, bacteria, and certain blood cells [41]. Notably, no antimicrobial peptide (AMP) genes were detected in our transcriptome-sequencing analysis. This is similar to the results of the genomic studies on *Acyrthosiphon pisum* [17], *D. citri* [42], and *B. tabaci* [43], and our results support the suggestion that hemipteran insects have lost some traditional immune genes during evolution. In addition, in fruit flies, silkworms, and other model organisms, the stimulation of the Toll and IMD pathways promotes the synthesis of a high concentration of AMPs [44,45]. However, in *C. chinensis*, due to the lack of AMPs, these traditional immune signaling pathways may drive the immune system by inducing genes with similar functions, which requires further study.

The expression of most immune-related genes was altered after *B. bassiana* infection. However, the regulation of these genes was different in the summer-form and winter-form *C. chinensis.* When a non-self enters the insect, PRPs recognize this exogenous pathogen, which is essential for generating immune signals [16]. Dscam is an antibody-like PRP that has been identified only in insects and crustaceans [46]. Studies in *Drosophila melanogaster* and *Anopheles gambiae* showed that interference with *Dscam* affected phagocytosis efficiency and led to increased mortality exposure in pathogens [47,48]. Galectin, a protein family found widely in animals and fungi, can bind to endogenous or surface glycans of pathogens [49]. In addition, galectin has been shown to enhance insect immunity in *Aedes aegypti* by competitively binding to receptors of *Bacillus thuringiensis* Cry toxins [50]. Two other types of PRPs have also been identified in *C. chinensis*—namely, hemocytin and scavenger receptor*—*which, in addition to recognizing bacteria, also recognize fungal and even plasmodium infections. Many studies have shown that the expression of PRPS will increase after the invasion of exogenous pathogens [51,52]. However, pathogens also have some unique ways of escaping host immunity. Another major insecticidal fungus, *Metarhizium anisopliae*, can express Metarhizium collagen-like protein (MCL) to cover the cell surface and evade recognition by the host’s PRPs [53]. Although genomic studies have shown that *B. bassiana* does not have MCL, the *PRP* genes were downregulated in summer-form *C. chinensis* after infection with *B. bassiana* [54]. This suggests that *B. bassiana* suppresses the host’s immune recognition. It is worth noting that after the transition to the winter form, *C. chinensis* recovered the recognition of *B. bassiana*. The regulatory mechanism needs further study.

Cells form the foundation of the immune response. The density of hemocytes changes after pathogen infection, reflecting the insect’s resistance [55,56]. The hemocyte concentration of winter-form *C. chinensis* increased more than that of the summer form after *B. bassiana* infection. This suggests that the winter form may have stronger cellular immunity than the summer form. This hypothesis is also supported by the analysis of the gene transcription level. Actin is a conserved and abundant cytoskeletal protein. In addition to participating in cell division, phagocytosis, and cell signaling, it has also been proven to be a bacteria-binding protein and shows direct killing activity against *Escherichia coli* [57,58]. Here, three *Actin* genes were downregulated in the summer form and upregulated in the winter form. Cathepsin is a crucial cysteine protease in the endolysosomal system that promotes cell proliferation and apoptosis [59,60]. In insects, cathepsin has been shown to respond to bacterial and fungal infections, and research on *Bombyx mori* has shown that the absence of cathepsin can directly affect the expression of Toll and IMD pathway-related genes [61,62,63]. Here, we identified three *Cathepsin* genes that, like *Actin*, were downregulated in the summer form, clearly affecting the antimicrobial activity of this form. Genes with the same expression profile also included phagosome process-related genes (two *tubulin*, one *syntaxin*-7, and two *protein transport protein Sec61 subunit gamma* genes), endocytosis-related genes, and other genes involved in cellular immunity. In conclusion, in the face of *B. bassiana* infection, the cellular immune system of the winter form is rapidly activated, while that of the summer form is suppressed to a certain extent at the transcript level.

The activation of serine protease cascade pathways constitutes a critical component of the innate immune system, playing an essential role in coordinating both the melanization response and Toll pathway activation within humoral immunity mechanisms [64]. To avoid the damage caused by overimmunity, organisms have also evolved negative regulators to control immune responses within a limited time and space. Serpin, the largest class of serine/cysteine peptidase inhibitors, inhibits immune signaling to downstream pathways by covalently binding to SP [65,66]. In the summer form, the expression of most *serpins* increased after *B. bassiana* infection, while in the winter form, *serpin* expression remained unchanged or was downregulated. At the same time, *SP* and *Toll* were induced by *B. bassiana* in the winter form but were significantly downregulated in the summer form. Obviously, the immune signaling process in the summer form was inhibited by the upregulation of s*erpin* genes. According to several studies, *Pieris rapae*’s endoparasitoid wasp *Pteromalus puparum* can secrete serpin to suppress host immune responses [67,68]. The research on *Helicoverpa armigera* and *Ostrinia furnacalis* found that the virus AcMNPV evades the host immune system by inducing the high expression of host *serpin* [69,70]. This suggests that the upregulation of *serpin* in the summer form may represent an immune evasion strategy evolved by *B. bassiana* that induces the host inhibitor to block further transmission of immune signals. How *B. bassiana* induces host inhibitory factors requires further investigation. Moreover, inhibitory factors in pest immune systems may be ideal genetic targets for improving pest control efficiency.

Compared with the summer form, the winter form exhibited significant downregulation of metabolic pathways, possibly because the immune system of the winter form works better. When immunity occurs, organisms mobilize most of their energy for proliferation, movement, immune signal transmission, and effector production of immune cells [71,72]. Consequently, due to competition with the immune system, other life processes such as growth, reproduction, and lipid accumulation are delayed, leading to overall metabolic suppression [73]. Adenosine 5′-triphosphate (ATP) is a crucial currency for metabolic and signaling pathways in organisms [74]. Normally, cells produce enough ATP to sustain essential life processes through oxidative phosphorylation [75]. However, in *C. chinensis*, the expression of some oxidative phosphorylation-related genes was affected upon *B. bassiana* infection. There were differences in the response of the oxidative phosphorylation process to *B. bassiana* in the two phenotypes. In the winter form, only downregulated genes were significantly enriched in oxidative phosphorylation, while in the summer form, upregulated genes were also significantly enriched in oxidative phosphorylation. Obviously, the degree of inhibition of oxidative phosphorylation in the winter form is more significant than that in the summer form. Similarly, in the process of cancer cell expansion and stem cell differentiation, ATP metabolism is also re-adjusted; that is, under aerobic conditions, ATP is not generated through oxidative phosphorylation, but activated glycolysis, which usually occurs under anaerobic conditions, is used to obtain ATP. This aerobic glycolysis (AG) process is known as the Warburg effect [76,77,78,79]. The aerobic glycolysis process is conserved between insects and mammals [80]. When faced with infection, although less ATP is obtained through the AG process, due to the need for a rapid response, it generates ATP faster, better meeting the urgent energy demands of immune cells [73,81]. After *Streptococcus pneumoniae* infection, *D. macrophages* induce metabolic conversion to aerobic glycolysis to meet the energy requirements of effective antimicrobial defense [80]. 6-phosphofructokinase (PFK) is a crucial rate-limiting enzyme in the glycolytic key rate-limiting steps, determining the direction and rate of the reaction. Following *B. bassiana* infection, *PFK* expression was upregulated in the winter form, whereas it was strongly inhibited in the summer form. Additionally, two other rate-limiting enzyme genes—*pyruvate kinase* and *hexokinase-1*—as well as genes involved in the glycolytic pathway, exhibited similar phenotypic differences after *B. bassiana* infection. This suggests that, compared with the summer form, the winter form better mobilized the AG process to rapidly energize the immune system. While our study highlights transcriptomic linkages between the metabolic and immune systems, the regulatory mechanisms driving these phenotype-specific responses remain elusive. We propose two non-exclusive hypotheses: (1) hormonal regulation, particularly juvenile hormone (JH) and ecdysteroid signaling pathways, as known mediators of morphogenetic transition in insects, may directly modulate the immune/metabolic interplay [82,83]; (2) epigenetic modifications, such as DNA methylation or non-coding RNAs, might stabilize phenotype-specific transcriptional programs [84]. Future investigations could test these models through the quantification of hormonal titers and methylation landscapes across seasonal morphs.

The phenotype-specific vulnerabilities of *C. chinensis* provide a scientific foundation for its precision control: for the summer form, the development of immunosuppressive biopesticides and genetically engineered *B. bassiana* strains with enhanced infectivity can be prioritized; for the winter form, the synergistic application of *B. bassiana* with glycolysis inhibitors to block immune activation, combined with metabolic pathway interference techniques to suppress overwintering energy reserves, is proposed. Concurrently, phenotype-driven niche partitioning was observed: the summer form reduces immunological costs by preferentially colonizing physiologically optimal pear hosts, whereas the winter form exhibits heightened resistance and exploits alternative overwintering hosts. Accordingly, we recommend cultivating pear varieties with elevated phenolic compounds to exacerbate metabolic imbalances in summer morphs, alongside establishing overwintering host trap zones to precisely disrupt pest colonization dynamics. This integrated strategy enables sustainable *C. chinensis* management by seasonally combining biocontrol and ecological regulation, targeting biological vulnerabilities while coordinating population dynamics and host interactions, and establishing a dual theoretical/practical framework for pest control.

Phenotypic plasticity in response to seasonal variations is widespread among plants and animals [85,86]. This seasonal polyphenism is not only manifested in morphological differences such as color and size, but also in life-history and reproduction [87,88,89]. However, the immune system, being complex and dynamically changing, has not been sufficiently studied. A few studies have found that compared with the spring morphs of *Araschnia levana*, the summer morphs have lower activity of immunity-related enzymes, and the production of AMPs in response to *Pseudomonas entomophila* infection is ineffective [90,91]. Here, after fungal infection, the expression of genes related to immune recognition, cellular immunity, and immune signaling is significantly suppressed in the summer form. This indicates that the fungus has weakened the host’s resistance. In contrast, *C. chinensis* acquires stronger immune capabilities through phenotypic plasticity, with its metabolic system allocating more energy to the immune system, leading to higher survival rates when exposed to pathogens. The divergent immune/metabolic strategies between summer-form and winter-form *C. chinensis* reflect complex adaptations to seasonal environmental stressors, with this phenotypic plasticity likely enhancing species survival by optimizing resource allocation: the summer form prioritizes rapid reproduction and dispersal under favorable conditions, whereas the winter form redirects energy toward immune defense to sustain population viability during environmental adversity. This trade-off aligns with the life-history trade-off theory, which posits that organisms balance investments in immunity against other life-history traits under selective pressures [92]. The trade-off between the immune system and other life processes of *C. chinensis* in different seasons is the result of long-term adaptation to the environment and is one of the foundations for its successful expansion. Our study lays a foundation for understanding the immunological significance of seasonal polyphenism, enriches the knowledge of insect/pathogenic fungi interactions, and provides insights and data for improving biological control efficiency against *C. chinensis*.

## Figures and Tables

**Table 1:** Summary of transcriptome data from PacBio platform.

Type	Number
Total CCS	116,535
Average CCS length (bp)	203,132,661
Mean read length of CCS	1743
Undesired primer reads	18,534
Full-length non-chimeric (FLNC) reads	93,175
Full-length non-chimeric percentage (FLNC%)	79.95%
High-quality isoforms	35,503
Low-quality isoforms	5
Average consensus isoforms read length	1724
Full-length non-redundant transcripts (FLNRTs)	30,097

## Data Availability

The datasets generated in this study have been uploaded to the NCBI database with the accession number PRJNA1225935.

## Supplementary Materials

The following supporting information can be downloaded at https://www.mdpi.com/article/10.3390/insects16050541/s1. Figure S1: Images of hemocyte types from *C. chinensis*: (A) prohemocytes; (B) plasmatocytes; (C) oenocytoids; (D) granulocytes; (E) spherulocytes; (F) megakaryocytes; Figure S2: Validation of gene expression levels. Data are shown as the means of three replicates (*n* = 3); Table S1: The primers used in the current study; Table S2: *C. chinensis* immune and stress gene list; Table S3: KEGG classification of DEGs.
