# Supplementary material for: Morphotype-Specific Antifungal Defense in Cacopsylla chinensis Arises from Metabolic and Immune Network Restructuring
Source: Insects. 2025 May 20;16(5):541. doi: 10.3390/insects16050541 (PMC12112565; doi:10.3390/insects16050541)
Supplement: Supplementary file 1 [file insects-16-00541-s001.zip › insects-3576370-supplementary.pdf]

Appendices

Table S1. **The primers used in current study.**

**Primer names**	**Primer sequences (5′ to 3′)**	**Length of primer**	**PCR product length**
rpL45-F	CCCTGGACTTTGAACAGGAA	20	
rpL45-R	CTCGTGGATACCGCAAGATT	20	
hy_transcript_33106-*F*	AAGCACAGGTGGGCGGAGAG	20	111
hy_transcript_33106-*R*	GGTGCCAGAATCCTACGCCATAC	23	
hy_transcript_12030-*F*	CGCCGCCTCTTCCTCCTCTC	20	93
hy_transcript_12030-*R*	GCCTCGGGACAACAGAACCTTTC	23	
hy_transcript_1466-*F*	TGCTGCTCAACTGATGGAACCAC	23	119
hy_transcript_1466-*R*	CCGCAGAGGCAGATAAAGGTGTC	23	
hy_transcript_29520-*F*	CGACGCCAACATTTTCACACACG	23	103
hy_transcript_29520-*R*	GAATCGCTCGGGGTACGCATAAC	23	
hy_transcript_14955-*F*	CAAGGTCATGCAGGTGGGAATGG	23	119
hy_transcript_14955-*R*	AGGCGGTAGGATAGTCGTTGGC	22	
hy_transcript_1054-*F*	TCAACGGTCTCGCCCATTGTTTG	23	94
hy_transcript_1054-*R*	ATGATACCCTGGACGGACACACC	23	
hy_transcript_10265-*F*	TCGTGTGGGAGGTGCTGAAAATC	23	145
hy_transcript_10265-*R*	TGCACGGTCTGAAGAGTTCATGG	23	
hy_transcript_10191-*F*	TACTTGCCGCCCGTCTCTATGG	22	125
hy_transcript_10191-*R*	CGTTTCCTCCGCTTTCTCCTCAC	23	
hy_transcript_10133-*F*	GCCATTTACCGAACGGGACCATC	23	137
hy_transcript_10133-*R*	TCCGATCCCTTGTCCGTCAACTC	23	

Table S2. *C. chinensis* immune and stress gene list.

Class	Role in Insect Immunity	Gene name	Gene ID	Protein length	Full length
Recognition	Multiple recognition	Dscam	hy_transcript_28829	451	yes
Recognition	Multiple recognition	Dscam	hy_transcript_13484	262	yes
Recognition	Fungal and bacterial recognition	Galectin	hy_transcript_8231	482	3'
Recognition	Fungal and bacterial recognition	Galectin	hy_transcript_14059	265	yes
Recognition	Fungal and bacterial recognition	Galectin	hy_transcript_27438	324	yes
Recognition	Fungal and bacterial recognition	Galectin	hy_transcript_29254	89	yes
Recognition	Fungal and bacterial recognition	Galectin	hy_transcript_23578	97	yes
Recognition	Fungal and bacterial recognition	Galectin	hy_transcript_20641	301	yes
Recognition	Fungal and bacterial recognition	Galectin	hy_transcript_22154	426	yes
Recognition	Fungal and bacterial recognition	Galectin	hy_transcript_15846	450	yes
Recognition	Fungal and bacterial recognition	Galectin	hy_transcript_2835	418	yes
Recognition	Multiple recognition	Hemocytin	hy_transcript_33738	565	yes
Recognition	Multiple recognition	hemocytin	hy_transcript_10222	156	3'
Recognition	Multiple recognition	Hemocytin	hy_transcript_24400	768	yes
Recognition	Multiple recognition	Hemocytin	hy_transcript_27692	560	yes
Recognition	Bind to lipoproteins	Scavenger receptor	hy_transcript_21623	562	yes
Recognition	Bind to lipoproteins	Scavenger receptor	hy_transcript_26411	562	yes
Recognition	Bind to lipoproteins	Scavenger receptor	hy_transcript_33712	589	yes
Recognition	Bind to lipoproteins	Scavenger receptor	hy_transcript_19237	548	yes
Signaling	Melanization process	Phenoloxidase-activating enzyme	hy_transcript_13875	113	3'
Signaling	Melanization process	Phenoloxidase-activating enzyme	hy_transcript_35267	336	3'
Signaling	Melanization process	Phenoloxidase-activating enzyme	hy_transcript_28004	132	yes
Signaling	Melanization process	Phenoloxidase-activating enzyme	hy_transcript_11203	230	yes
Signaling	Melanization process	Phenoloxidase	hy_transcript_13741	424	yes
Signaling	Melanization process	Phenoloxidase	hy_transcript_438	364	yes
Signaling	Melanization process	Phenoloxidase	hy_transcript_446	714	yes
Signaling	Melanization process	Serpin	hy_transcript_10817	398	yes
Signaling	Melanization process	Serpin	hy_transcript_11624	385	yes
Signaling	Melanization process	Serpin	hy_transcript_12700	316	3'
Signaling	Melanization process	Serpin	hy_transcript_13953	184	3'
Signaling	Melanization process	Serpin	hy_transcript_14173	413	yes
Signaling	Melanization process	Serpin	hy_transcript_19467	385	yes
Signaling	Melanization process	Serpin	hy_transcript_19943	385	yes
Signaling	Melanization process	Serpin	hy_transcript_20134	281	3'
Signaling	Melanization process	Serpin	hy_transcript_22874	417	yes
Signaling	Melanization process	Serpin	hy_transcript_2299	410	yes
Signaling	Melanization process	Serpin	hy_transcript_24746	410	yes

Signaling	Melanization process	Serpin	hy_transcript_2594	385	yes
Signaling	Melanization process	Serpin	hy_transcript_27250	413	yes
Signaling	Melanization process	Serpin	hy_transcript_2784	410	yes
Signaling	Melanization process	Serpin	hy_transcript_30034	385	yes
Signaling	Melanization process	Serpin	hy_transcript_32733	528	yes
Signaling	Melanization process	Serpin	hy_transcript_3334	339	3'
Signaling	Melanization process	Serpin	hy_transcript_34721	371	Yes
Signaling	Melanization process	Serpin	hy_transcript_34906	186	Yes
Signaling	Melanization process	Serpin	hy_transcript_501	335	Yes
Signaling	Melanization process	Serpin	hy_transcript_6893	399	3'
Signaling	Melanization process	Serpin	hy_transcript_8018	385	Yes
Signaling	Melanization process	Serpin	hy_transcript_8057	142	3'
Signaling	Melanization process	Serpin	hy_transcript_9016	230	Yes
Signaling	Melanization process	Serpin	hy_transcript_9560	283	3'
Signaling	Toll pathway	Spaetzle-processing enzyme	hy_transcript_35337	128	Yes
Signaling	Toll pathway	Spätzle	hy_transcript_32694	166	Yes
Signaling	Toll pathway	toll-like receptor	hy_transcript_22332	311	Yes
Signaling	Toll pathway	Cactus	hy_transcript_20395	422	Yes
Signaling	Toll pathway	Traf	hy_transcript_14408	250	Yes
Signaling	Imd pathway	Iap	hy_transcript_10024	517	3'
Signaling	Imd pathway	Iap	hy_transcript_3351	276	3'
Signaling	Imd pathway	Tak	hy_transcript_16203	456	Yes
Signaling	Jak/stat pathway	Pias	hy_transcript_20642	436	Yes
Signaling	Jnk pathway	Kayak	hy_transcript_26832	254	Yes
Signaling	Jnk pathway	Eiger	hy_transcript_10481	570	3'
Signaling	Eicosanoid signaling pathway	iPL A2	hy_transcript_21345	452	3'
Signaling	Eicosanoid signaling pathway	iPL A2	hy_transcript_9487	352	Yes
Signaling	Eicosanoid signaling pathway	iPL A2	hy_transcript_34696	174	Yes
Signaling	Eicosanoid signaling pathway	sPL A2	hy_transcript_19110	205	Yes
Response	Microbial degradation	Lysozyme	hy_transcript_22655	218	3'
Response	Microbial degradation	Lysozyme	hy_transcript_23084	183	Yes
Response	Microbial degradation	Lysozyme	hy_transcript_23402	92	Yes
Response	Microbial degradation	Lysozyme	hy_transcript_3937	213	3'
Response	Microbial degradation	Lysozyme	hy_transcript_28918	156	Yes
Response	Microbial degradation	Lysozyme	hy_transcript_30341	179	Yes
Response	Scavenging free radical	Superoxide dismutase	hy_transcript_21854	178	Yes
Response	Scavenging free radica	Superoxide dismutase	hy_transcript_19575	217	Yes
Response	Scavenging free radica	Superoxide dismutase	hy_transcript_35099	155	Yes
Response	Scavenging free radica	Superoxide dismutase	hy_transcript_31655	155	Yes
Response	Scavenging free radica	Superoxide dismutase	hy_transcript_3962	155	Yes
Response	Scavenging free radica	Superoxide dismutase	hy_transcript_4423	217	Yes
Response	Scavenging free radica	Superoxide dismutase	hy_transcript_17850	188	Yes
Response	Scavenging free radica	Superoxide dismutase	hy_transcript_26892	218	Yes

Response	Scavenging free radica	Peroxidase	hy_transcript_10759	586	Yes
Response	Scavenging free radica	Peroxidase	hy_transcript_1098	652	Yes
Response	Scavenging free radica	Peroxidase	hy_transcript_12832	396	3'
Response	Scavenging free radica	Peroxidase	hy_transcript_26252	548	Yes
Response	Scavenging free radica	Peroxidase	hy_transcript_33067	767	Yes
Response	Scavenging free radica	Peroxidase	hy_transcript_6845	369	Yes
Response	Scavenging free radica	Catalase	hy_transcript_20386	500	Yes
Response	Scavenging free radica	Catalase	hy_transcript_18227	500	Yes
Response	Scavenging free radica	Catalase	hy_transcript_34413	500	Yes
Response	Scavenging free radica	Thioredoxin reductase	hy_transcript_10191	494	Yes
Response	Scavenging free radica	Thioredoxin	hy_transcript_14018	137	Yes
Response	Scavenging free radica	Thioredoxin	hy_transcript_27400	101	Yes
Response	Scavenging free radica	Peroxiredoxin	hy_transcript_15123	221	Yes
Response	Scavenging free radica	Peroxiredoxin	hy_transcript_12821	220	Yes
Response	Scavenging free radica	Peroxiredoxin	hy_transcript_1648	156	Yes
Response	Scavenging free radica	Peroxiredoxin	hy_transcript_19689	182	Yes
Response	Scavenging free radica	Peroxiredoxin	hy_transcript_26871	190	Yes
Response	Scavenging free radica	Peroxiredoxin	hy_transcript_22344	178	Yes
Response	Scavenging free radica	Peroxiredoxin	hy_transcript_31644	196	Yes
Response	Scavenging free radica	Peroxiredoxin	hy_transcript_3832	196	Yes
Response	Scavenging free radica	Peroxiredoxin	hy_transcript_21033	190	Yes
Response	Scavenging free radica	Peroxiredoxin	hy_transcript_2748	227	Yes
Response	Scavenging free radica	Peroxiredoxin	hy_transcript_14530	178	Yes
Response	Scavenging free radica	Peroxiredoxin	hy_transcript_18044	179	Yes
Response	Scavenging free radica	Peroxiredoxin	hy_transcript_18688	178	Yes
Response	Scavenging free radica	Peroxiredoxin	hy_transcript_18556	412	3'
Response	Detoxification	Glutathione S-transferase	hy_transcript_12809	205	Yes
Response	Detoxification	Glutathione S-transferase	hy_transcript_19796	216	Yes
Response	Detoxification	Glutathione S-transferase	hy_transcript_2821	249	3'
Response	Detoxification	Glutathione S-transferase	hy_transcript_8798	221	Yes
Response	Detoxification	Glutathione S-transferase	hy_transcript_2258	216	Yes
Response	Detoxification	Glutathione S-transferase	hy_transcript_30331	192	3'
Response	Detoxification	Cytochrome P450	hy_transcript_1183	566	Yes
Response	Detoxification	Cytochrome P450	hy_transcript_1210	603	3'
Response	Detoxification	Cytochrome P450	hy_transcript_1499	563	Yes
Response	Detoxification	Cytochrome P450	hy_transcript_13237	211	3'
Response	Detoxification	Cytochrome P450	hy_transcript_16107	687	Yes
Response	Detoxification	Cytochrome P450	hy_transcript_12565	449	Yes

Response	Detoxification	Cytochrome P450	hy_transcript_19825	514	Yes
Response	Detoxification	Cytochrome P450	hy_transcript_10572	241	Yes
Response	Detoxification	Cytochrome P450	hy_transcript_14033	513	Yes
Response	Fungal degradation	Chitinase-like protein	hy_transcript_10069	197	Yes
Response	Fungal degradation	Chitinase-like protein	hy_transcript_1321	436	Yes
Response	Fungal degradation	Chitinase-like protein	hy_transcript_19485	402	Yes
Response	Fungal degradation	Chitinase-like protein	hy_transcript_11053	301	Yes
Response	Fungal degradation	Chitinase-like protein	hy_transcript_28693	237	Yes

The mark of Yes, 5' and 3' means that the fragment of the unigene consists of complete open reading frame, 5' -end containing start codon and 3' -end containing stop codon, respectively.

Table S3. KEGG classification of DEGs.

KEGG main category	KEGG subcategory	Summer form		Winter form	
		Gene Count	KO Count	gene Count	KO Count
Cellular Processes		979	305	267	100
	Cell growth and death	63	41	9	7
	Cell motility	12	7	2	2
	Cellular community - eukaryotes	23	14	6	6
	Transport and catabolism	916	266	255	90
Environmental Information Processing		483	226	122	51
	Membrane transport	30	15	47	25
	Signal transduction	410	186	67	20
	Signaling molecules and interaction	46	27	11	9
Metabolism		2880	830	959	363
	Amino acid metabolism	477	155	155	65
	Biosynthesis of other secondary metabolites	3	2	0	0
	Carbohydrate metabolism	967	222	371	105
	Energy metabolism	675	183	208	97
	Global and overview maps	832	175	321	102
	Glycan biosynthesis and metabolism	131	69	19	15
	Lipid metabolism	405	132	101	53
	Metabolism of cofactors and vitamins	358	116	137	49
	Metabolism of other amino acids	275	67	90	29
	Metabolism of terpenoids and polyketides	39	24	5	3
	Nucleotide metabolism	176	65	72	33
	Xenobiotics biodegradation and metabolism	147	31	36	18
Organismal Systems		340	112	108	43
	Aging	111	27	38	11
	Circulatory system	26	9	8	3
	Development and regeneration	37	15	7	4

	Digestive system	18	9	11	8
	Endocrine system	61	20	32	15
	Environmental adaptation	4	4	2	2
	Excretory system	1	1	1	1
	Immune system	49	28	18	13
	Nervous system	3	2	0	0
	Sensory system	63	14	18	6
Genetic Information Processing		2372	833	601	256
	Folding, sorting and degradation	783	285	190	80
	Replication and repair	89	59	9	6
	Transcription	348	143	68	28
	Translation	1289	392	374	156
Human Diseases		80	17	29	25
	Cancer: overview	24	12	7	7
	Cancer: specific types	4	3	1	1
	Cardiovascular disease	4	2	3	3
	Drug resistance: antineoplastic	1	1	0	0
	Endocrine and metabolic disease	12	6	9	6
	Immune disease	4	3	0	0
	Infectious disease: bacterial	20	12	8	7
	Infectious disease: parasitic	11	5	10	6
	Infectious disease: viral	26	14	11	10
	Neurodegenerative disease	12	7	11	10
	Substance dependence	3	2	0	0

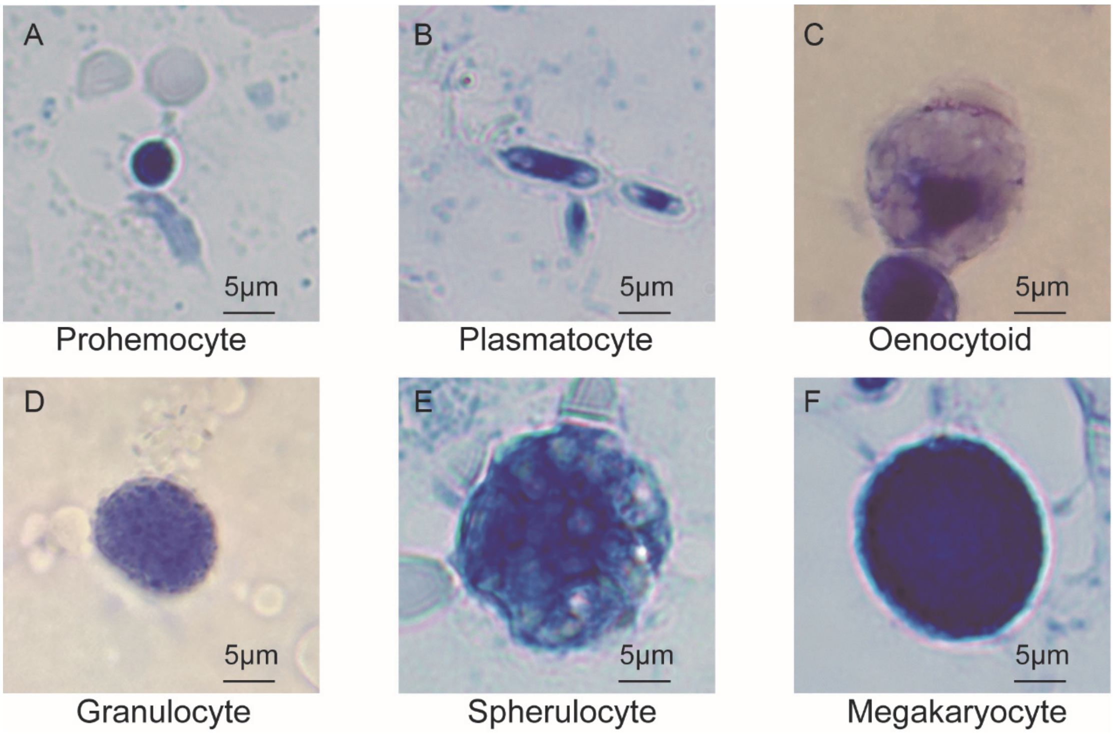

Figure S1. Images of hemocyte types from *C. chinensis*: (A) prohemocytes; (B) plasmatocytes; (C) oenocytoids; (D) granulocytes; (E) spherulocytes; (F) megakaryocytes.

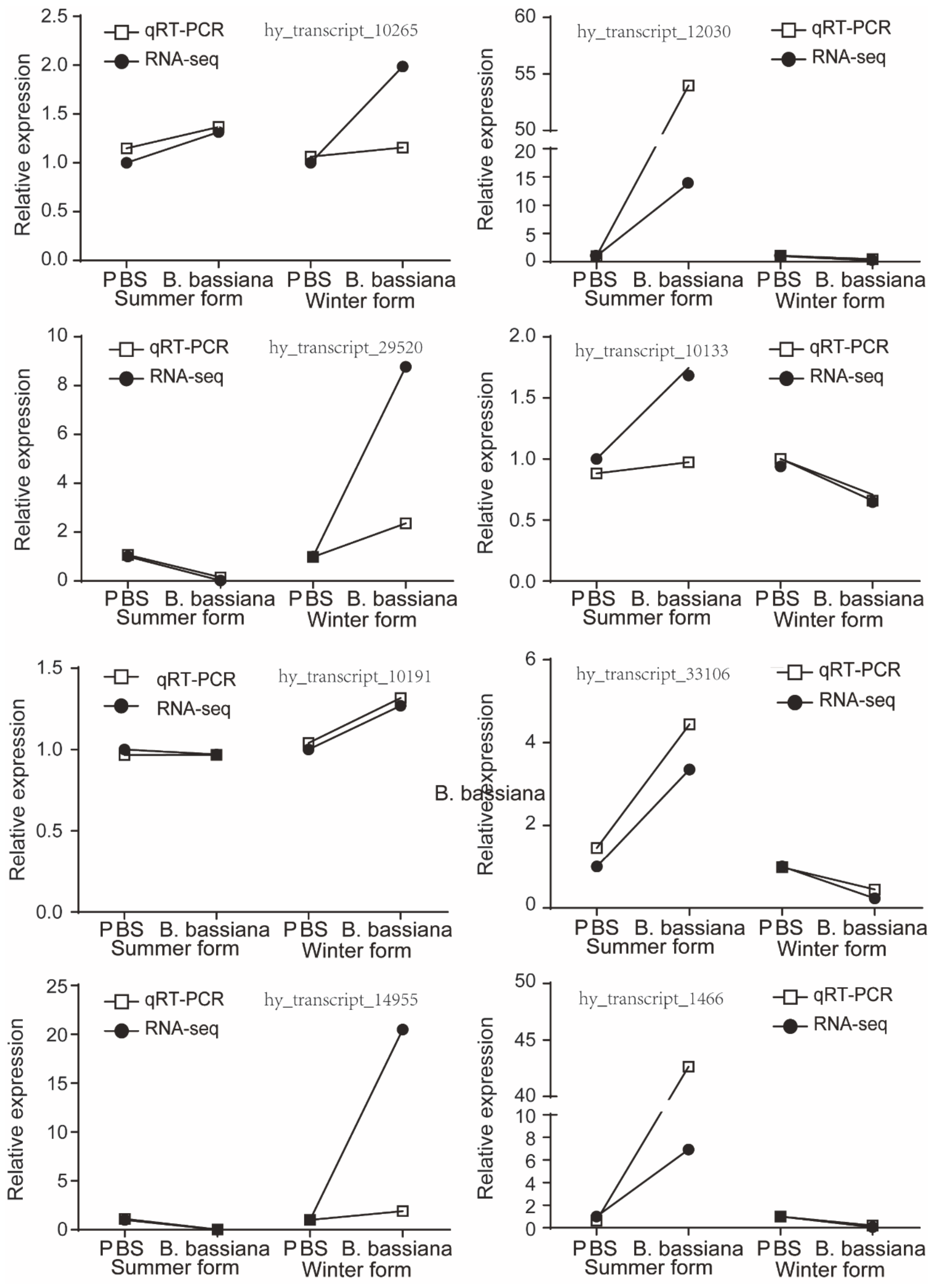

Figure. S2. Validation of gene expression levels. Data are shown as the mean of three replicates (n = 3).
